# Interactions between Polygenetic Variants and Lifestyle Factors in Hypothyroidism: A Hospital-Based Cohort Study

**DOI:** 10.3390/nu15173850

**Published:** 2023-09-03

**Authors:** Da Sol Kim, Sunmin Park

**Affiliations:** Department of Food and Nutrition, Obesity/Diabetes Research Center, Hoseo University, Asan 31499, Republic of Korea; tpfptm14@daum.net

**Keywords:** hypothyroidism, white blood cell counts, immunity, inflammation, diet pattern

## Abstract

Hypothyroidism is a prevalent endocrine disorder and is associated with a variety of metabolic disturbances. This study aimed to investigate the polygenic variants associated with hypothyroidism risk and the interaction of polygenic risk scores (PRS) with dietary patterns in influencing disease risk in 56,664 participants aged >40 in a hospital-based cohort. The participants were classified as having hypothyroidism (n = 870) diagnosed by a physician and no hypothyroidism (n = 55,794). Genetic variants associated with hypothyroidism were identified using a genome-wide association study (GWAS). Genetic variants interacting with each other were selected using a generalized multifactor dimensionality reduction analysis, and the PRS generated was evaluated for interaction with lifestyle parameters. Coffee, alcohol, meat intake, and a Korean balanced diet were inversely associated with hypothyroidism risk, as were selenium, copper, and manganese intakes. White blood cell (WBC) counts and serum alkaline phosphatase and triglyceride concentrations were positively associated with hypothyroidism risk, as were osteoporosis and thyroid cancer. The GMDR analysis generated a three-single nucleotide polymorphism (SNP) model comprising dual oxidase-1 (*DUOX1*)_rs1648314; thyroid-stimulating hormone receptor (*TSHR*)_rs75664963; and major histocompatibility complex, class-II, DQ Alpha-1 (*HLA-DQA1*)_rs17426593. The PRS derived from the three- and seven-SNP models were associated with a 2.11- and 2.32-fold increase in hypothyroidism risk, respectively. Furthermore, the PRS from the three-SNP model showed interactions with WBC counts, wherein the positive association with hypothyroidism risk was more pronounced in participants with low WBC counts than those with high WBC counts (≥4 × 10^9^ /L). Dietary patterns, such as the plant-based diet (PBD) and the Western-style diet (WSD), along with smoking status, exhibited interactions with the PRS, influencing hypothyroidism risk. In participants with a high PRS, those in the high-PBD, low-WSD, and smoker groups had a higher proportion of hypothyroidism than those in the low-PBD, high-WSD, and non-smoker groups. In conclusion, genetic variants related to immunity and thyroid hormone secretion were linked to hypothyroidism risk, and their PRS interacted with PBD and WSD intake and smoking status. These results contribute to a better understanding of hypothyroidism and its prevention strategies for precision medicine intervention.

## 1. Introduction

Thyroid hormone production is tightly controlled by the hypothalamus–pituitary–thyroid (HPT) axis. When triiodothyronine (T3) and thyroxine (T4) levels are sufficient, they exert negative feedback on the hypothalamus and pituitary, reducing the release of thyrotropin-releasing hormone (TRH) and thyroid-stimulating hormone (TSH), respectively, to normalize T3 and T4 levels [1]. The disruption of the HPT axis elevates the TSH concentrations above the reference level and free thyroxine concentration below the reference range, leading to hypothyroidism. With a prevalence of approximately 5% in the general population, primary hypothyroidism accounts for over 99% of hypothyroid cases [1]. Hypothyroidism is more common in women and tends to increase with age [2]. Primary hypothyroidism typically results from damage to the thyroid gland itself, often due to autoimmune conditions such as Hashimoto’s thyroiditis, iodine deficiency, certain medications, and prior thyroid surgery or radiation in persons having sufficient iodine intake [3]. The thyroid hormone regulates energy metabolism, body temperature, heart rate, and overall metabolism. Inadequate thyroid hormone levels lead to fatigue, weight gain, sensitivity to cold, dry skin, and cognitive impairment [4,5].

While the etiology of hypothyroidism is multifaceted and often complex, genetic factors have emerged as significant contributors to disease susceptibility. Recent genetic studies have shed light on the role of polygenic variants, which result from multiple genetic alterations across various loci, in influencing an individual’s risk of developing hypothyroidism, particularly congenital hypothyroidism [6]. Genetic mutations associated with hypothyroidism are related to thyroid dysgenesis-related genes, such as *thyroid-stimulating hormone receptor (TSHR)*, *Forkhead Box E1 (FOXE1)*, *NK2 Homeobox 1 (NKX2-1)*, *Paired* Box 8 *(PAX8)*, and *NK2 Homeobox 5 (NKX2-5)*, as well as thyroid dyshormonogenesis linked genes, such as *Solute Carrier Family 5 Member 5 (SLC5A5)*, *Thyroid Peroxidase (TPO)*, *Dual oxidase 2 (DUOX2)*, *Dual oxidase maturation factor 2 (DUOXA2)*, *Solute Carrier Family 6 Member 4 (SLC6A4)*, and *iodothyronine dehalogenase (DEHAL1)* [6,7]. These genes are linked to essential thyroid functions, including thyroid differentiation, iodide organification, thyroglobulin synthesis, iodide transport, and iodotyrosine deiodination [6,8]. Understanding these genetic predispositions can offer valuable insights into disease pathogenesis, enabling early detection and personalized treatment approaches.

In addition to genetics, environmental factors have a crucial impact on thyroid hormone production, secretion, and function, contributing to the development of hypothyroidism [9]. These environmental influences can directly affect the thyroid gland or interfere with the production and regulation of TSH and thyroxin [9]. The factors include iodine deficiency, exposure to toxins, radiation, chronic stress, psychological factors affecting the HPT axis, consumption of certain goitrogenic foods (e.g., cabbage, broccoli, and cauliflower), exposure to endocrine-disrupting chemicals found in plastics and pesticides, and triggers related to autoimmune conditions such as viral infections and specific pollutants [10]. Despite the significance of these environmental factors, few studies have explored the relationship between hypothyroidism and dietary patterns. A review paper suggested that the Mediterranean diet may improve thyroid function [11]. However, the interaction between genetic variants and dietary intake in hypothyroidism remains largely unexplored.

Understanding the complex interplay between genetic susceptibility to hypothyroidism and the influence of dietary choices is vital for advancing our knowledge of disease pathogenesis. This study aimed to investigate the association of polygenic variants with hypothyroidism risk and the interaction of polygenic risk scores (PRS) with dietary patterns and lifestyle factors influencing disease risk. The findings of this research could potentially lead to the development of targeted and individualized approaches to managing and mitigating the risk of hypothyroidism.

## 2. Methods

### 2.1. Participants

This present study recruited 58,701 volunteers aged between 40 and 79 years for a cohort that included multiple hospitals as part of the Korean Genome and Epidemiology Study (KoGES) during 2010–2014 [8]. The institutional review boards (IRB) of the Korea National Institute of Health and Hoseo University approved the KoGES (KBP-2015-055 and 1041231-150811-HR-034-01, respectively). All participants signed a written informed consent form. 

### 2.2. Baseline Characteristics, Anthropometric, and Biochemical Parameters of the Participants

General characteristics, including age, gender, education, income, smoking status, alcohol consumption, and physical activity, were surveyed on the initial visit [12,13]. Smoking status was categorized into non-smokers, past smokers who had not smoked for the last six months, and current smokers who had smoked at least 20 cigarettes in their lifetime [14]. The amount of alcohol intake was calculated by multiplying the alcohol content consumed on each occasion by frequency. Coffee intake was also measured in the same manner [14]. Regular physical activity was assessed by asking if the subjects engaged in 150 min or more of physical activity per week, and the response was marked “Yes” or “No”. 

Height and weight were obtained at the initial interview as previously described, and body mass index (BMI) was calculated by body weight (kg) divided by squares of height (m^2^). Obesity was defined as BMI ≥ 25 kg/m^2^. Blood pressure was determined by a physician using a sphygmomanometer in a sitting position under resting conditions three times, and the average systolic blood pressure (SBP) and diastolic blood pressure (DBP) were used. Blood was collected in a fasting state, and lipid profiles, alanine aminotransferase (ALT), aspartate aminotransferase (AST), alkaline phosphatase (ALP), and creatinine in the serum were measured using a Hitachi 7600 Automatic Analyzer (Hitachi, Tokyo, Japan). Plasma glucose and blood glycosylated hemoglobin (HbA1c) levels and white blood cell (WBC) count were measured using an automatic analyzer (ZEUS 9.9; Takeda, Tokyo, Japan). Serum C-reactive protein (CRP) concentrations were measured using an enzyme-linked immunosorbent assay (ELISA) kit. 

### 2.3. Definition of Hypothyroidism and Metabolic Syndrome 

Those participants diagnosed with hypothyroidism by a physician were considered to have the disease. The subjects with the experience of hyperthyroidism, cancers, chronic kidney disease, and brain-related diseases such as dementia and Parkinson’s disease (n = 2037) were excluded from the study. The participants with and without hypothyroidism were 870 and 55,794, respectively. Metabolic syndrome (MetS) was defined as having three or more of the following traits: abdominal obesity measured by waist circumference, hyperglycemia, hypertension, hypo–high density lipoprotein (HDL) cholesterolemia, and hypertriglyceridemia, including conditions for which the individual may be taking medication. This definition was per the 2005 revised National Cholesterol Education Program Adult Treatment Panel III (NCEP-ATP III) criteria for Asia [15,16].

### 2.4. Estimation of Usual Food Intake by a Semi-Quantitative Food Frequency Questionnaire (SQFFQ) 

An SQFFQ was designed to assess the usual food consumption of Koreans, and the accuracy and reproducibility were validated [17]. The SQFFQ included 106 food items Koreans consume as a typical diet. During the last year, the food intake was scored as more than, equal to, or less than the standard portion size in grams that was visualized using photographs of each food. Food frequencies were divided into the following nine categories: never or seldom, once per month, two to three times monthly, once or twice weekly, three or four times weekly, five or six times weekly, daily, twice daily, and ≥3 times daily. The daily food intakes were estimated by multiplying the median of the daily consumption frequencies by the portion size for each food category. The nutrients in the food intake per the SQFFQ were calculated using the Can-Pro 2.0 nutrient intake assessment software developed by the Korean Nutrition Society (Seoul, Republic of Korea). The dietary inflammation index was calculated by multiplying the inflammatory scores of 38 food components and nutrients reported in the previous study. However, garlic, ginger, saffron, and turmeric were excluded from the dietary inflammation index formula since their intake was not recorded in the KoGES study. The sums of the scores of 34 items were divided by 100, as described previously [18].

### 2.5. Dietary Patterns by Principal Component Analysis

Dietary pattern analysis was conducted based on the consumption of 30 predefined food groups from 106 food items in the SQFFQ, as previously reported [19]. Dietary patterns were generated using the pre-categorized food groups’ principal component analysis (PCA). The number of clusters was made based on eigenvalues > 1.5, and four dietary patterns were generated [20]. The orthogonal rotation procedure (varimax) yielded four uncorrelated dietary patterns. Foods with ≥0.40 factor-loading values were considered to have a predominant contribution to the specific pattern [21]. The four dietary patterns were the Korean balanced diet (KBD), plant-based diet (PBD), Western-style diet (WSD), and rice-based diet (RBD) (Supplemental Table S1). 

### 2.6. Quality Control of Genotyping and GWAS for Hypothyroidism Risk

The genotypes of the 58,701 participants were provided by the Center for Genome Science, Korea National Institute of Health. The genotyping of genomic DNA extracted from whole blood was performed using the Affymetrix Genome-Wide Human single nucleotide polymorphism (SNP) Array 5.0 (Affymetrix, Santa Clara, CA, USA). Genotyping quality and accuracy were determined using the Mahalanobis Distance genotyping algorithm with Bayesian robust linear modeling (BRLM) [18]. The genotype inclusion criteria were as follows: ≥98% genotyping accuracies, <4% rate of missing genotype calls, ≤30% heterozygosity, no gender biases, and *p* > 0.05 Hardy–Weinberg equilibrium (HWE) [18]. 

**Figure 1:**
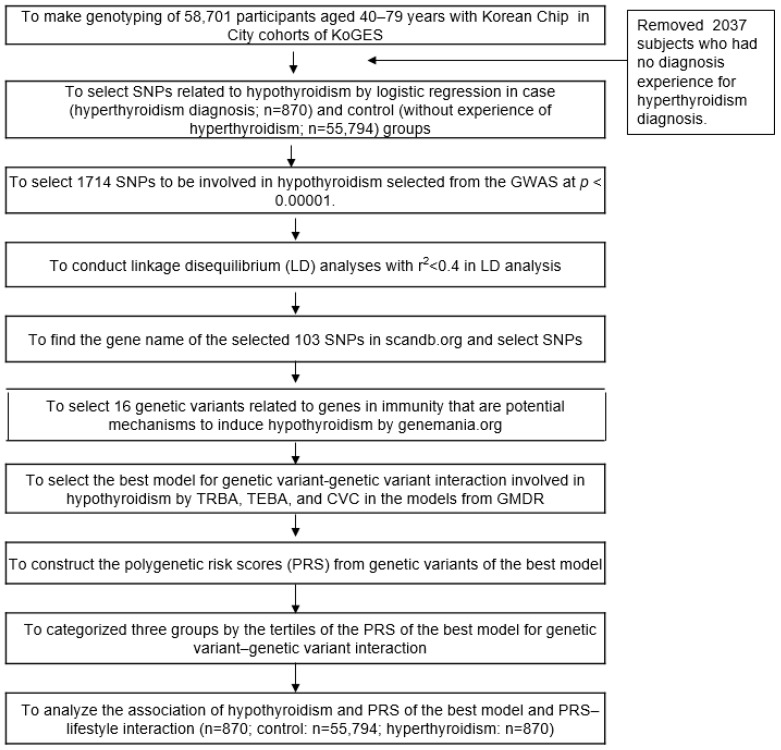
Flow chart to generate the polygenic risk score system influencing hypothyroidism risk.

The genetic variant selection for hypothyroidism is present in Figure 1. The genetic association of hypothyroidism was investigated using genome-wide association studies (GWAS) after adjusting for age, gender, residence area, income, BMI, energy and alcohol intake, physical exercise, and smoking using PLINK (http://pngu.mgh.harvard.edu/~purcell/plink accessed on 10 January 2023). The distribution and appropriateness of genetic variants were checked using Manhattan and Quantile–Quantile (Q–Q) plots. The lambda value of the Q–Q plot was close to 1, indicating the appropriateness of the GWAS results. Among the genetic variants of the GWAS, those with high linkage disequilibrium (LD) (D′ < 0.2, r^2^ > 0.05) were excluded using Haploview 4.2 in PLINK since they provided the same genetic information for hypothyroidism risk [22].

### 2.7. Genetic Variant–Genetic Variant Interaction by a Generalized Multifactor Dimensionality Reduction (GMDR) Method 

GMDR is a nonparametric and genetic model designed to detect and characterize nonlinear interactions between discrete genetic attributes. We applied the GMDR method to find the interacting genetic variants associated with hypothyroidism risk. The criteria for selecting the optimal genetic model were a significant *p*-value (*p* < 0.05) for the sign test of trained balance accuracy (TRBA) and test balance accuracy (TEBA) and a cross-validation consistency (CVC) score of 9 or 10 out of 10 [22]. The PRS was calculated by summing the number of the risk alleles in the genetic variants of the selected optimal model. 

### 2.8. Statistical Analysis

Statistical analysis was performed using SAS (version 9.3; SAS Institute, Cary, NC, USA). The PRS of the selected model were categorized into three groups (low, middle, and high). The frequency distributions of the categorical variables were examined by applying the Chi-square test. A one-way analysis of variance (ANOVA) was conducted for the continuous variables among the PRS groups after adjusting for age, sex, BMI, education, income, energy intake, alcohol intake, smoking, and physical activity. 

The association of hypothyroidism risk with anthropometric, biochemical, and genetic parameters was evaluated using adjusted logistic regression analysis. According to covariates, the first model was analyzed with adjustments for the area of residence, gender, age, and BMI, and the second model included the covariates in model 1 plus energy intake, smoking and drinking status, total physical activity, medication for asthma, and energy intake. The odds ratios (ORs) and 95% confidence intervals (CIs) were assessed using adjusted logistic regression using the low-PRS as a reference.

The potential interaction between the PRS and lifestyle factors for hypothyroidism risk was conducted with a multivariate general linear model (GLM) analysis with the main effects of PRS and lifestyle, their interaction, and covariates. Lifestyles were categorized into two groups (low and high levels) with the proper cutoff. An adjusted logistic regression analysis was performed in two groups based on the cutoff values assigned to each lifestyle parameter. The proper cutoff values for the two groups were assigned for each variable. This classification was based on the assumption that the low level of each parameter had a higher likelihood of interacting with the PRS. The specific cutoff values for each parameter were used with recommended intake for nutrients and 33rd percentiles of some parameters, such as dietary inflammation index and dietary patterns, and the exact values were found in the table legend. Based on the classification criteria, participants were then categorized into the high and low groups of lifestyle parameters. A p-value of ≤ 0.05 was considered statistically significant. 

## 3. Results

### 3.1. Demographic Characteristics and Nutrient Intake of Participants

The incidence of hypothyroidism in the study population was 1.48% (n = 870) (Table 1). Most participants with hypothyroidism were female; women had an 8.3-fold higher risk of hypothyroidism. Smoking status was not associated with hypothyroidism risk, but drinking was inversely associated with the risk of hypothyroidism (Table 1). The proportion of participants with hypothyroidism was higher in the exercise group than in the non-exercise group, but physical exercise was not significantly related to hypothyroidism risk. Intake of alcohol and coffee was lower in the participants with hypothyroidism than in healthy participants but only in women, and these intakes were inversely associated with hypothyroidism risk (Table 1). Energy intake did not differ between the healthy and the hypothyroid groups. Intake of carbohydrates, fats, proteins, and fiber did not differ in those with and without hypothyroidism. Iodine intake, one of the risk factors for hypothyroidism, did not vary between the healthy and hypothyroidism groups. Intake of antioxidant minerals, such as selenium, copper, and manganese, was much lower in the hypothyroidism group than the healthy group and was inversely associated with hypothyroidism (Table 1). However, the intake of antioxidant vitamin C did not differ between the two groups. Vitamin D intake and the dietary inflammation index did not differ between the two groups (Table 1). 

Seaweed, vegetable, and fruit intake did not differ between the healthy and hypothyroidism groups (Table 1). Meat intake was lower in the hypothyroidism group than in the healthy group and was inversely linked to hypothyroidism risk. The proportion of participants with hypothyroidism was much lower in the high-KBD than in the low-KBD groups, and KBD was inversely associated with hypothyroidism risk (Table 1). However, PBD, WSD, and RMD were not associated with hypothyroidism risk (Table 1). 

### 3.2. Anthropometric and Biochemical Measurements

BMI, waist and hip circumferences, plasma glucose, and blood HbA1c levels did not differ between the healthy and hypothyroidism groups and were not associated with its risk (Table 2). Serum total cholesterol and triglyceride concentrations were higher in the hypothyroidism group. The serum triglyceride concentrations were positively associated with hypothyroidism risk. The estimated glomerular filtration rate (eGFR, renal function index) and serum ALT and AST concentrations (liver function index) did not differ significantly between the two groups (Table 2). Interestingly, the serum ALP concentrations were much higher in the hypothyroidism group than in the healthy group, but only in women, and it was positively associated (2.11-fold) with hypothyroidism risk. There was a 3.15-fold increase in the risk of hypothyroidism in participants with <4.0 × 10^9^ /L WBC count (Table 2). However, this association was not seen in those with serum CRP concentrations. Cancer incidence was higher in the hypothyroidism group than in the healthy group, and hypothyroidism risk was 1.93 times higher in those with cancer (Table 2). The cancers, primarily thyroid cancer, raised the risk of hypothyroidism 3.16 times. The incidence of osteoporosis and arthritis was higher in the hypothyroidism group than in the healthy group and was positively associated with hypothyroidism risk (Table 2).

### 3.3. Genetic Variants Associated with Hypothyroidism Risk by GWAS and SNP–SNP Interactions by GMDR

The statistical significance of the genetic variants associated with hypothyroidism is shown in a Manhattan plot (Supplementary Figure S1A). Since the number of genetic variants with *p* < 5 × 10^−8^ was not sufficient to find proper genetic variants for hypothyroid risk, a liberal significance level (*p* < 5 × 10^−5^) was applied. Lambda, a genome inflation factor of genetic variants for hypothyroidism risk, was at 1.064, indicating no inflation of the genetic variants (Supplementary Figure S1B). 

Among the genetic variants associated with hypothyroidism risk, ten genetic variants were selected with GMDR. The genetic characteristics of selected genetic variants (*p* < 5 × 10^−5^) are presented in Table 3. Three SNPs were positively associated (OR > 1), and seven SNPs were inversely associated with hyperthyroidism risk (0 < OR < 1). Two SNPs located in chromosome 6, including *Major Histocompatibility Complex*, *Class II*, *DQ Alpha 1 (HLA-DQA1)*, and *Chromosome 6 Open Reading Frame 15 (C6orf15)* and one SNP, *HORMA Domain Containing 2 (HORMAD2*), in chromosome 22 were associated with immunity. One SNP in chromosome 8, *tumor necrosis factor receptor superfamily*, *member 11b (TNFRSF11B)*, was linked to inflammation (Table 3). Two SNPs, *Dual oxidase-1 (DUOX1)* and *Dual oxidase-2 (DUOX2)*, were associated with modulating oxidative stress in the thyroid, and *TSHR* was related to thyroid secretion. The minor allele frequency (MAF) of the ten SNPs was between 0.026 and 0.326. The p-value of the HWE was >0.05.

After conducting the GMDR, the optimal SNP–SNP interaction models contained three and seven genetic variants. The three-SNP model included *DUOX1*_rs1648314, *TSHR*_rs75664963, and *HLA-DQA1*_rs17426593. The seven-SNP model contained the SNPs in the three-SNP model plus *Sirtuin 3(SIRT3)* rs11246015, *Vav Guanine Nucleotide Exchange Factor 3 (VAV3)*_rs4915077, *TNFRSF11B*_rs1157, and *C6orf15*_rs2233955 (Supplementary Table S1). These three- and seven-SNP models had a *p*-value < 0.05 of the sign test of trained balanced accuracy (TRBA) and test balance accuracy (TEBA) after adjusting for age, gender, seaweed intake, and BMI for covariate set 1 and energy intake, physical activity, alcohol intake, and smoking status plus covariate set 1 for covariate set 2. The cross-validation consistency (CVC) of both models was 10/10 (Supplementary Table S1). These results indicated that the three- and seven-SNP models with SNP–SNP interactions contribute to the risk of hyperthyroidism.

### 3.4. Association between Polygenic Risk Scores (PRS) and Hypothyroidism Risk

**Figure 2:**
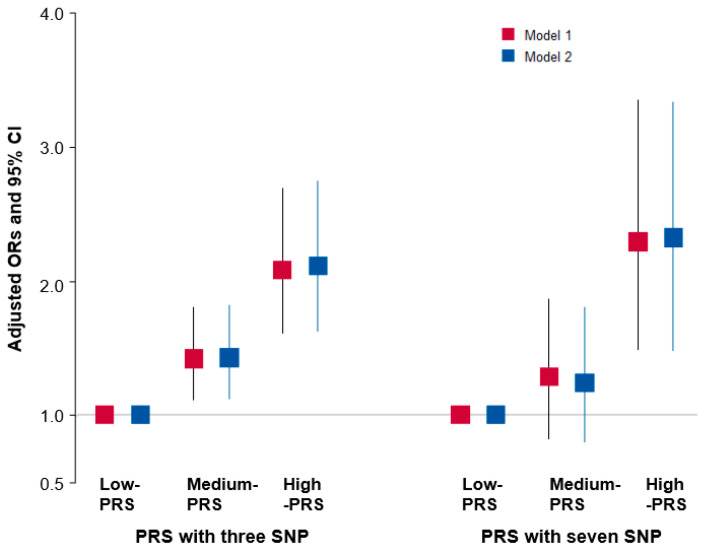
Adjusted odds ratio (ORs) and 95% confidence intervals (CIs) of the polygenic risk scores (PRS) of three- and seven-single nucleotide polymorphism (SNP) models generated for assessing SNP–SNP interactions associated with hypothyroidism risk. The best generalized multifactor dimensionality reduction analysis (GMDR) models with three-SNPs and seven-SNPs were calculated by summing the number of risk alleles of six and seven SNPs. The calculated PRS were divided into three categories (0–3, 4–5, and ≥6; 0–5, 6–8, and ≥9), the low-PRS, medium-PRS, and high-PRS groups, for the three-SNP and seven-SNP models, respectively. The adjusted OR was analyzed by logistic regression with covariates, including age, gender, residence areas, income, education, energy intake, smoking status, physical activity, alcohol intake, and the survey year. The reference group was the low-PRS in logistic regression. Red and blue boxes indicate the adjusted ORs for the three and seven SNPs, respectively, and the lines through red and blue boxes indicate 95% CIs.

The PRS was calculated from the three- or seven-SNP models by the GMDR models, and it was categorized into three groups: low-, medium-, and high-PRS. A high-PRS exhibited a 2.11 (1.63–2.74)- and 2.32 (1.48–3.33)-times higher hypothyroidism risk, respectively, compared with a low-PRS in the three-SNP and seven-SNP models after adjusting for the covariate set 2 (Figure 2). The adjusted ORs and 95% CI in the three-SNP and seven-SNP models were similar after adjustment of covariate sets 1 and 2. The PRS of the three-SNP model was associated with WBC counts and asthma but not serum CRP concentration and MetS-related biochemical parameters (Supplementary Table S2). 

### 3.5. Genetic Interactions of Lifestyle Factors with Hypothyroidism Risk 

**Figure 3:**
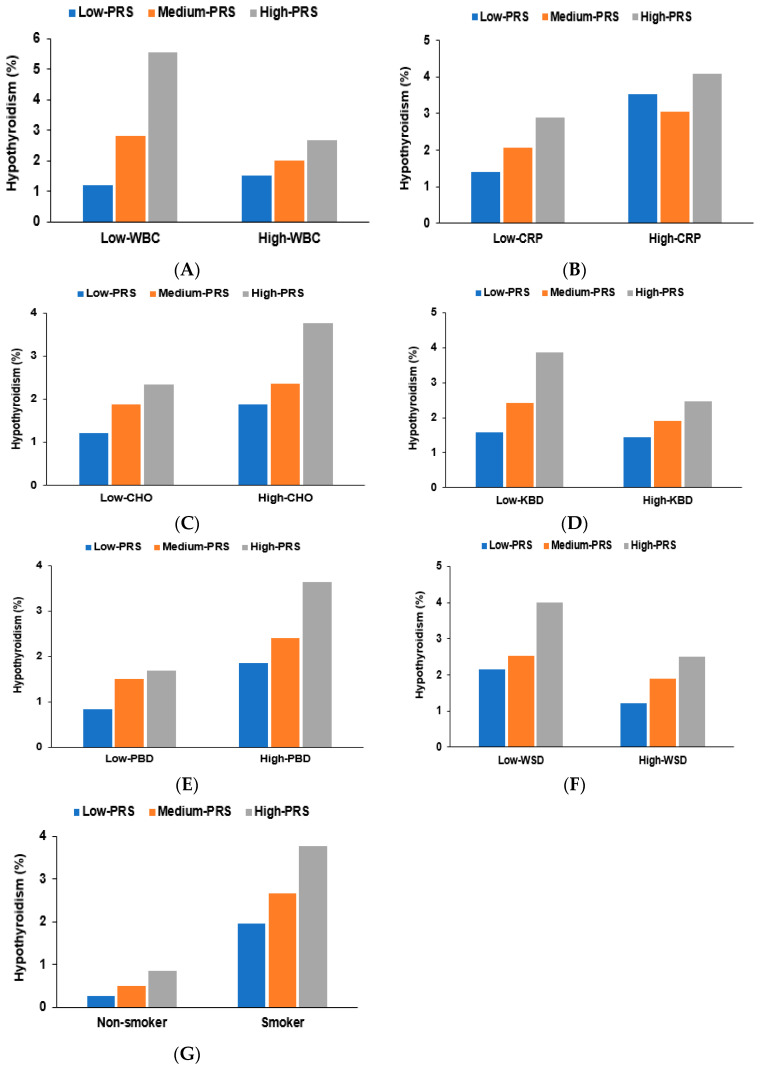
The proportion of individuals with hypothyroidism with the polygenic risk scores (PRS) of the three-single nucleotide polymorphism (SNP) model according to white blood cell (WBC) counts, diets, and smoking status. (**A**). WBC (cutoff: 4.0 × 10^9^ /L); (**B**). Serum C-reactive protein (CRP) concentration (Cutoff: 0.5 mg/dL); (**C**). Carbohydrate intake (Cutoff: 70th energy percentile); (**D**). Korean balanced diet (KBD, Cutoff: 33rd percentile); (**E**). Plant-based diet (PBD, Cutoff: 33rd percentile); (**F**). Western-style diet (WSD, Cutoff: 33rd percentile); (**G**). Smoking status; Low-PRS (0–3), Medium-PRS (4–5), and High-PRS (≥6) in the six-SNP model.

There was an interaction between the WBC count and the PRS from the three-SNP model to influence hypothyroidism risk. A high PRS increased the risk of hypothyroidism 4.89-fold in the low WBC count group and 1.71-fold in the high WBC count group (Figure 3A). The proportion of participants with hypothyroidism was much higher in the low WBC count than in the high WBC count group. The high-PRS group included more participants with hypothyroidism than the low-PRS group, regardless of WBC count. However, the serum CRP concentration did not influence hypothyroidism risk. The proportion of participants with hypothyroidism was much higher in the high CRP group than in the low CRP group (Figure 3B). The proportion of participants with hypothyroidism was higher in the high-PRS than the low-PRS group within the low CRP group. However, there was no significant difference among the PRS groups within the high CRP group (Table 4). 

Energy and macronutrient intakes did not interact with the PRS and did not contribute to hypothyroidism risk. However, carbohydrate intake was close to significant in the interaction with PRS (*p* = 0.056). The proportion of participants with hypothyroidism was much higher in those with a high carbohydrate intake than in those with a low intake (Figure 3C). The proportion of participants with hypothyroidism increased with the PRS, which was much higher in the low-KBD than in the high-KBD groups (Figure 3D). The PBD and WSD interacted with PRS to influence hypothyroidism risk among the different dietary patterns. The proportion of participants with hypothyroidism increased with the PRS, which was much higher in the high-PBD than in the low-PBD group (Figure 3E). KBD showed a trend similar to that of the WSD but WSD showed a pattern opposite to that of PBD. The proportion was higher in those with a low WSD intake than those with a high WSD intake (Figure 3F). Among the lifestyle factors, physical activity, coffee, and alcohol intake did not interact with the PRS to contribute to hypothyroidism risk. However, smoking interacted with PRS to influence hypothyroidism risk (Figure 3G). The proportion of participants with hypothyroidism increased with PRS in both smokers and non-smokers but was much higher in smokers than in non-smokers in each PRS group.

## 4. Discussion

This present study highlights the association between genetic variants and hypothyroidism risk in a hospital-based cohort of participants aged over 40 years. Among the 56,664 participants, 870 were diagnosed with hypothyroidism, while 55,794 did not have hypothyroidism. This study identified several genetic variants related to immunity and the thyroid hormone secretion associated with hypothyroidism. The PRS derived from these genetic variants were positively correlated with hypothyroidism risk. Furthermore, the PRS showed interactions with lifestyle factors, such as dietary patterns (PBD and WSD) and smoking status, influencing hypothyroidism risk. These findings provide valuable insight into the genetic basis and potential preventive strategies for hypothyroidism. 

Hypothyroidism exhibits distinct gender-related patterns in its prevalence. It is generally more common in females than males [23], consistent with the findings of the present study. Estrogen is a significant factor influencing thyroid function, and its interaction with various factors makes the relationship complex. However, the exact mechanisms through which estrogen affects the thyroid are still under investigation. Estrogen has been linked to the production, release, and metabolism of thyroid hormones from the thyroid gland. It modulates the synthesis of TSH in the pituitary gland [24,25]. Estrogen can also impact the levels of thyroid-binding proteins in the bloodstream, which, in turn, affects the transport and availability of thyroid hormones for cellular uptake and metabolism [26]. Moreover, elevated estrogen levels have been positively associated with developing autoimmune thyroid disorders, such as Hashimoto’s thyroiditis [25]. Hormonal fluctuations at different life stages, such as puberty, pregnancy, and menopause, can impact thyroid function differently among individuals, potentially contributing to an increased risk of thyroid disorders in females [24,27]. 

Hypothyroidism is primarily a condition of severe dietary iodine deficiency called goiter. In case there is a sufficient intake of seaweed and iodine-fortified salt, the primary cause of hypothyroidism is thyroiditis due to an autoimmune disorder (Hashimoto’s thyroiditis) and thyroidectomy or radioactive iodine therapy [1]. In different studies, the mean iodine intake of Koreans varies between 200 and 550 ug/day, mainly from seaweed intake (66%), milk and dairy products (11%), and fish (9%) [28,29]. This present study showed an intake of about 325 ug/day for men and 445 ug/day for women, which was higher than the recommended intake (150 ug/day) but lower than 2400 ug/day, the upper limit of iodine in Korea DRI. Previous studies have reported that excess iodine intake is associated with hyperthyroidism or hypothyroidism [30,31]. In Koreans, the risk of non-immune-related hypothyroidism is significantly elevated with excess iodine intake (≥750 ug/day) with a hazard ratio (HR) of 2.81 times (1.64–4.80) [32]. Individuals with predisposing thyroid diseases, such as autoimmune thyroiditis or thyroidectomy, are susceptible to iodine-induced hypothyroidism when they consume iodine-fortified salt and drinking water [30]. However, this present study did not show a significant difference in iodine intake between the hypothyroidism and healthy groups. Therefore, hypothyroidism is not related to iodine intake in Koreans whose intake is adequate. 

Moderate alcohol consumption serves as a protective factor against various autoimmune diseases, including overt autoimmune hypothyroidism [33,34]. Aligning with prior research, this current study establishes an inverse connection between alcohol consumption and hypothyroidism. However, the precise mechanism behind this effect remains undisclosed. The impact of coffee consumption on hypothyroidism remains uncertain. Analysis of NHANES data from 2007 to 2012 reveals that the incidence of subclinical hypothyroidism is lower when coffee intake is <2 cups per day compared to ≥2 cups per day, and it tends to be lower in the <2 cups per day group than in those who abstain from coffee [35]. In contrast, the participants in our study exhibited significantly lower coffee intake compared to NHANES participants, and this diminished coffee consumption showed an inverse correlation with hypothyroidism. The relationship between coffee intake and hypothyroidism necessitates further investigation.

Thyroid hormones cause the breakdown of carbohydrates and lipids for energy production and regulate the synthesis and breakdown of glucose, cholesterol, and triglycerides. It is suggested that thyroid hormones have multiple effects on glucose and lipid metabolism and energy consumption and play an essential role in MetS development. These hormones are also involved in heart function, the central nervous system, bone growth and turnover, and menstrual cycle and fertility in women. It has been observed in earlier studies that due to decreased thermogenesis and metabolic rates, individuals with hypothyroidism were more obese than healthy adults [36,37]. However, this present study showed that the BMI and waist circumferences were not significantly different between individuals with and without hypothyroidism. Furthermore, type 2 diabetes mellitus reduces TSH levels, impairs the conversion of T4 to T3 in the peripheral tissues, and is positively associated with hypothyroidism [38]. However, no association was observed between serum glucose concentration and hypothyroidism in this present study. Hypothyroidism has been reported to be linked to lipid profiles, but this remains controversial [39,40]. This present study showed that serum total cholesterol, HDL, and LDL concentrations were not associated with hypothyroidism, but serum triglyceride concentration was positively associated with hypothyroidism. Therefore, the MetS status was not linked to hypothyroidism risk in this present study. 

Thyroid hormones have multiple effects on glucose and lipid metabolism and energy consumption and play an essential role in the development of MetS. Diseases such as thyroid cancer, osteoporosis, and arthritis are positively associated with hypothyroidism risk. WBC count indicates the immune status, and a low WBC count raises the risk of hypothyroidism 3.15-fold, indicating that hypothyroidism might be linked to autoimmune diseases. This might account for the higher incidence of most cancers, particularly thyroid cancer, in individuals with hypothyroidism. Previous studies have supported the association of cancer incidence with hypothyroidism risk. However, these results are inconsistent [41]: Breast cancer and radiation therapy to the supraclavicular lymph nodes are linked to hypothyroidism risk [42]. Elevated TSH, increased reactive oxygen species production, and mutation/polymorphisms of genes have been involved in increased cancer risk or pro-tumoral cell behavior [43]. Therefore, there is a need for more clinical studies in subclinical or clinical hypothyroidism to explore the relationship between lifestyle modification and hypothyroidism prevention. 

The relationship between nutrient and diet intake and hypothyroidism remains unclear. Selenium and iodine are essential for thyroid hormone production and function, as their intake protects the thyroid gland from free radicals during thyroid hormone synthesis [44]. An increase in oxidative stress results in an oxidant/antioxidant imbalance in individuals with hypothyroidism [45,46]. A deficiency in selenium, zinc, and magnesium intake can significantly impact hypothyroidism risk. In a meta-analysis of 32 observational studies, individuals with hypothyroidism were observed to have lower serum selenium and zinc levels than healthy adults. [47]. A 10-week zinc, vitamin A, and magnesium supplementation intervention increases serum-free T4 concentration and prevents the increase of serum CRP and malondialdehyde levels and body weight in adults with hypothyroidism [48]. Selenium and zinc supplementation has been found to be beneficial in specific populations with otherwise limited generalizability [49]. This present study found that the intakes of selenium, copper, manganese, and zinc, which function as cofactors of antioxidant enzymes, were inversely associated with hypothyroidism risk, suggesting that hypothyroidism is linked to oxidative stress. However, the DII, vitamin C, vitamin D, and fiber intakes were not linked to hypothyroidism risk. Meat intake was inversely associated with hypothyroidism risk, but seaweed, vegetable, and fruit intakes were not associated with hypothyroidism. Furthermore, KBD was inversely associated, and PBD was positively associated with hypothyroidism risk. Therefore, maintaining a well-balanced diet that includes a variety of nutrient-rich foods is essential for supporting overall health and thyroid function in individuals with hypothyroidism.

Autoimmune activation can induce both hypothyroidism and hyperthyroidism, which result from different mechanisms within the autoimmune process [50]. Over time, the autoimmune attack leads to destroying thyroid tissue, reducing the gland’s ability to produce thyroid hormones (T3 and T4) and inducing hypothyroidism [51]. However, in Graves’ disease, a common autoimmune cause of hyperthyroidism, the immune system produces thyroid-stimulating immunoglobulins (TSI) that mimic the action of the TSH [51,52]. TSI binds to the TSH receptors on thyroid cells, stimulating the thyroid gland to overproduce thyroid hormones [52]. Therefore, both hypothyroidism and hyperthyroidism are associated with autoimmune disorders, and they are linked to immune and inflammation-related genetic variants. This present study showed that *HLA-DQA1_*rs17426593 and *TNFRSF11B_*rs11573856 were associated with hypothyroidism, suggesting a link between hypothyroidism, autoimmune disorders, and inflammation. 

Thyroid hormone secretion is regulated by TSH acting via the TSH receptor (TSHR), a G protein-coupled transmembrane receptor. TSHR mediates thyroid hormone synthesis in the thyroid gland and height growth [53]. The TSHR loss-of-function mutation contributes to blocking TSH action to increase TSH levels to induce hypothyroidism with thyroid hypoplasia [53]. However, Graves’ disease stimulates the production of TSHR antibodies, leading to the development of hyperthyroidism [52]. TSHR mutations are also reported to be associated with Graves’ disease [51]. According to the location of mutation sites on the *TSHR* gene, hypothyroidism or hyperthyroidism may be induced. However, no *TSHR* mutation has been found for hyperthyroidism in the city hospital-based cohort of KoGES [50]. In Chinese patients with congenital hypothyroidism, seven genetic variants of *TSHR*, such as mutations in Ile216, Ala275, Asn372, and Ser567 with loss-of-function, and genetic variants of *DUOX2* are also found [54,55]. Therefore, the *TSHR* mutation may be mainly linked to hypothyroidism risk in Asians. 

Notably, no previous studies have reported interactions between genetic variants and lifestyle factors and the association with hypothyroidism, making this present study unique and groundbreaking in its approach. This present study showed the interaction of genetic impact by PRS with PBD and WSD, contributing to hypothyroidism risk. Interestingly, the proportion of individuals with hypothyroidism increased with PRS and was much higher in the high PBD than in the low PBD groups. The trend of hypothyroidism with PRS in the PBD group was opposite to those in the WSD and KBD groups. The proportion of individuals with hypothyroidism was much higher in the low KBD and low WSD groups than in the high KBD and high WSD groups, regardless of the PRS. Smoking status had an interaction with the PRS to influence hypothyroidism risk. Overall, this study’s findings demonstrated the intricate relationship between genetic factors, dietary patterns, and smoking status in the context of hypothyroidism risk. Identifying these interactions adds significant value to our existing knowledge and highlights the importance of considering genetic and lifestyle factors in understanding and managing hypothyroidism. Further research is needed to explore the underlying mechanisms and clinical implications of these interactions for developing personalized preventive strategies and targeted interventions for individuals at risk of hypothyroidism. 

In this present study, a novel finding was the relationship of the PRS for hypothyroidism with immunity and thyroid function and its interaction with dietary patterns. However, the limitations of this study may be summarized as follows: (1) The data were derived from a cross-sectional study. Therefore, causal relationships could not be established. (2) Serum TSH, T3, and T4 concentrations were not measured, and the participants were classified as having hypothyroidism based on a question regarding any previous diagnosis of hypothyroidism. Misclassification or measurement errors in these variables could introduce bias into the observed associations. (3) The usual food intake was calculated from the SQFFQ containing 106 foods designed for Korean meals, which was validated with 3-day food records for four seasons. However, the SQFFQ has some measurement bias in measuring food intake. 4) The genetic variants were not validated in an independent cohort since other cohorts available in Korea did not include hypothyroidism-related data. However, the number of participants was large enough to enhance the statistical power for conducting robust analyses and increasing the generalizability of the results. 

In conclusion, this hospital-based cohort study provides valuable insights into the genetic basis and potential preventive strategies for hypothyroidism. This study identified genetic variants related to immunity and thyroid hormone secretion that are associated with hypothyroidism risk. The PRS derived from these genetic variants was positively correlated with hypothyroidism risk, highlighting the importance of genetic factors in disease susceptibility. Moreover, this study demonstrated that the PRS interacts with certain lifestyle factors, specifically PBD and WSD, as well as smoking status, influencing hypothyroidism risk. Notably, the interaction of PRS with the PBD and WSD leads to contrasting trends in hypothyroidism risk, depending on the dietary patterns followed by individuals. These findings emphasize the intricate interplay between genetic and lifestyle factors in determining hypothyroidism risk, thus adding novel insights to our understanding of the condition.

## Figures and Tables

**Table 1:** Demographic characteristics and nutrient intake of the participants according to hyperthyroidism.

	Men		Women		Adjusted ORs and 95% CI
	Normal (n = 19,970)	Hypothyroidism (n = 60)	Normal (n = 35,824)	Hypothyroidism (n = 810)	
Age (years)	54.8 ± 0.07 ^a^	57.1 ± 1.23 ^a^	49.6 ± 0.24 ^b^	51.6 ± 1.71 ^a^**^+++^	1.444 (1.243–1.678)
Gender (%)	35.8	6.9	64.2	93.1 ***	8.276 (6.294–10.88)
Hypothyroidism treatment (N, % treatment)	-	49 (81.7)	-	609 (75.2)	
Former+current Smoking (Number, %)	5604 (28.1)	14 (23.3)	702 (1.97)	13 (1.60)	0.859 (0.605–1.220)
Drinking (>20 g/day)	14,262 (71.6)	39 (65.0)	10,879 (30.5)	191 (23.6) ***	0.716 (0.603–0.851)
Coffee (>1 c/weeks)	13,510 (67.7)	41 (68.3)	21,292 (59.4)	444 (54.8) **	0.859 (0.74–0.990)
Physical activity (N, Yes%)	8822 (45.5)	33 (55.0)	13,426 (38.5)	354 (43.8) **	1.135 (0.986–1.306)
Energy (EER%) ^1^	84.6 ± 0.05 ^c^	84.3 ± 0.69 ^c^	102 ± 0.03 ^b^	103 ± 0.19 ^a+++^	0.980 (0.853–1.127)
CHO (En%) ^2^	60.9 ± 0.07 ^b^	60.6 ± 1.09 ^b^	72.5 ± 0.05 ^a^	73.1 ± 0.29 ^a+++^	1.152 (0.952-1.393)
Fat (En%) ^3^	11.6 ± 0.05 ^b^	11.4 ± 0.72 ^b^	15.0 ± 0.03 ^a^	15.0 ± 0.19 ^a^***	0.516 (0.253–1.052)
Protein (En%) ^4^	11.1 ± 0.02 ^b^	11.3 ± 0.37 ^b^	14.1 ± 0.02 ^a^	14.1 ± 0.10 ^a+++^	0.952 (0.779–1.164)
Fiber (g/day) ^5^	5.59 ± 0.02 ^b^	5.69 ± 0.34 ^ab^	6.04 ± 0.07 ^a^	6.08 ± 0.47 ^ab++^	0.851 (0.703–1.031)
Iodine (ug/day) ^6^	329 ± 2.8 ^b^	317 ± 39.7 ^b^	442 ± 1.89 ^a^	450 ± 10.6 ^a+++^	0.943 (0.809–1.100)
Selenium ^7^	17.3 ± 0.19 ^a^	11.1 ± 2.71 ^ab^	13.0 ± 0.13 ^b^	11.4 ± 0.72 ^b^**	0.737 (0.616–0.882)
Cu ^8^	0.93 ± 0.01 ^a^	0.80 ± 0.10 ^a^	0.87 ± 0.01 ^a^	0.79 ± 0.03 ^b^*	0.766 (0.642–0.914)
Mn ^9^	2.27 ± 0.02 ^a^	1.77 ± 0.21 ^b^	2.08 ± 0.01 ^a^	1.88 ± 0.06 ^b^**	0.743 (0.633–0.872)
Zn ^10^	4.74 ± 0.03	3.96 ± 0.40	4.72 ± 0.02	4.38 ± 0.11 **	0.810 (0.688–0.954)
Vitamin C ^11^	91.4 ± 0.52 ^b^	94.0 ± 7.45 ^b^	113 ± 0.35 ^a^	110 ± 1.99 ^a+++^	1.053 (0.905–1.226)
Vitamin D ^12^	5.21 ± 0.05 ^b^	5.07 ± 0.66 ^b^	7.07 ± 0.03 ^a^	7.17 ± 0.18 ^a+++^	1.050 (0.883–1.249)
Dietary inflammation index ^13^	−18.5 ± 0.13 ^b^	−19.1 ± 1.85 ^ab^	−20.7 ± 0.09 ^a^	−20.3+0.49 ^a^	0.961 (0.812–1.137)
Sodium ^14^	2.41 ± 0.01	2.39 ± 0.15	2.44 ± 0.01	2.35 ± +0.04	1.011 (0.871–1.174)
Seaweeds (g/day) ^15^	1.66 ± 0.01 ^b^	1.89 ± 0.27 ^b^	2.45 ± 0.05 ^a^	2.39 ± 0.37 ^ab++^	0.868 (0.739–1.020)
Vegetables (g/day) ^16^	89.1 ± 0.81 ^b^	99.4 ± 11.5 ^ab^	114 ± 0.54 ^a^	115 ± 3.07 ^a+^	0.973 (0.834–1.135)
Fruits (g/day) ^17^	167 ± 1.9 ^b^	165 ± 26.5 ^b^	243 ± 1.3 ^a^	240 ± 7.1 ^a++^	1.080 (0.921–1.266)
Meats (g/day) ^18^	86.7 ± 0.88 ^a^	79.5 ± 12.5 ^ab^	83.1 ± 0.60 ^a^	75.0 ± 3.35 ^b^*	0.803 (0.674–0.956) *
Traditional balanced diet ^19^	14,661 (73.4)	47 (78.3)	22,151 (61.8)	458 (56.5) **	0.848 (0.731–0.984)
Prudent diet ^19^	8943 (44.8)	28 (46.7)	26,455 (73.9)	636 (78.5) **	1.242 (1.045–1.476)
Western-style diet ^19^	15,640 (78.3)	45 (75.0)	22,888 (63.9)	493 (60.9)	0.923 (0.794–1.073)
Rice-based diet ^19^	12,790 (64.1)	37 (61.7)	23,848 (66.6)	513 (63.3)	0.877 (0.756–1.018)

Values represent adjusted means, standard errors, adjusted odds ratio (ORs), and 95% confidence intervals (CI) after adjusting for covariates of age, BMI, residence area, income, education, smoking and drinking status, and physical activity. In analyzing adjusted ORs, each parameter was divided into two groups with the cutoff, which was estimated energy requirement (EER) ^1^, 70 energy percent (En%) ^2^, 14 En% ^3^, 15 en% ^4^, 4 g/d ^5^, 461 ug/d ^6^, 15 ug/d ^7^, 1 ug/d ^8^, 2.1 ug/d ^9^, 5 ug/d ^10^, 100 mg/d ^11^, 10 ug/d ^12^; 66th percentile ^13^, 2.0 g/d ^14^, 0.6 ug/d ^15^, 1 g/d ^16^, 2 g/d ^16^, 295 g/d ^17^, 76 g/d ^18^; and 33th percentile ^19^. Meats = meat + chicken + processed meat. Participants were divided into four groups according to hypothyroidism and gender. ^a, b^ Different superscript letters indicated significantly different at *p* < 0.05. * Significantly different by hypothyroidism at *p* < 0.05, ** at *p* < 0.01, and *** at *p* < 0.001. ^+^ Significantly different by genders at *p* < 0.05, ^++^ at *p* < 0.01, and ^+++^ at *p* < 0.001.

**Table 2:** Adjusted means of the metabolic parameters according to gender and hypothyroidism.

	Men		Women		Adjusted ORs (95% CI)
	Normal (n = 19,970)	Hypothyroidism (n = 60)	Normal (n = 35,824)	Hypothyroidism (n = 810)	
BMI (kg/m^2^) ^1^	24.5 ± 0.02 ^a^	24.3 ± 0.45 ^a^	23.7 ± 0.09 ^b^	23.5 ± 0.62 ^a+#^	0.896 (0.765–1.048)
Waist circumference (cm) ^2^	85.7 ± 0.04 ^a^	86.2 ± 0.74 ^a^	80.5 ± 0.15 ^b^	79.8 ± 1.04 ^b+++^	0.850 (0.680–1.063)
Hip circumference (cm) ^3^ 98	95.7 ± 0.04 ^a^	97.0 ± 0.67 ^a^	94.4 ± 0.13 ^b^	93.6 ± 0.93 ^ab+++#^	1.007 (0.818–1.238)
Fasting plasma glucose (mg/dl) ^4^ 126	100 ± 0.2 ^a^	99.0 ± 3.8 ^ab^	97.1 ± 0.78 ^b^	93.7 ± 5.33 ^ab^	0.981 (0.740–1.300)
HbA1c (%) ^5^ 6.5	5.81 ± 0.01	5.75 ± 0.16	5.80 ± 0.04	5.61 ± 0.21	0.949 (0.667–1.348)
Total-C (mg/dl) ^6^ 230	193 ± 0.3 ^b^	195 ± 5.8 ^b^	201 ± 1.2 ^a^	213 ± 8.1 ^ab+^	1.102 (0.939–1.292)
LDL-C (mg/dl) ^7^ 160	113 ± 0.3 ^b^	112 ± 4.5 ^b^	122 ± 0.2 ^a^	118 ± 1.2 ^ab++^	1.142 (0.956–1.365)
HDL-C (mg/dl) ^8^	49.1 ± 0.1 ^b^	50.9 ± 1.9 ^b^	56.9 ± 0.4 ^a^	57.8 ± 0.5 ^ab+++^	0.967 (0.831–1.126)
TG (mg/dl) ^9^	155 ± 0.9 ^a^	159 ± 16.9 ^ab^	125 ± 3.4 ^b^	112 ± 23.6 ^b++^	1.274 (1.089–1.490) **
SBP (mmHg) ^10^	125 ± 0.1 ^a^	123 ± 2.2 ^ab^	120 ± 0.4 ^b^	120 ± 3.1 ^ab+^	0.878 (0.748–1.030)
DBP (mmHg) ^11^	78.3 ± 0.1 ^a^	77.0 ± 1.5 ^b^	74.1 ± 0.3 ^b^	71.9 ± 2.1 ^b+++^	0.751 (0.530–1.002)
MetS (N, Yes%)	16,430 (82.3)	47 (78.3)	31,433 (87.7)	713 (88.0)	1.078 (0.861–1.350)
eGFR ^12^ (mL/min/1.73m^2^)	83.9 ± 0.14 ^b^	86.6 ± 2.05 ab	87.3 ± 0.10 ^a^	87.9 ± 0.55 ^a+^	0.862 0.693 1.074)
Serum ALT (U/L) ^12^	26.7 ± 0.16 ^a^	24.4 ± 3.0 ^ab^	21.8 ± 0.61 ^b^	22.4 ± 4.16 ^ab^	0.856 (0.651–1.127)
Serum AST (U/L) ^13^	25.7 ± 0.12 ^a^	23.9 ± 2.23 ^ab^	23.6 ± 0.46 ^b^	25.2 ± 3.12 ^ab^	1.090 (0.761–1.562)
Serum ALP (U/L) ^14^	186 ± 1.2 ^a^	172 ± 18.3 ^ab^	167 ± 4.2 ^b^	189 ± 25.3 ^ab^***	2.110 (1.820–2.445)
WBC (×10^9^ /L) ^15^ 4.0	5.80 ± 0.02 ^a^	5.60 ± 0.20 ^a^	5.65 ± 0.01 ^ab^	5.55 ± 0.05 ^b+^	3.151 (2.601–3.817) ***
Serum CRP (mg/dL) ^16^ 0.5	0.17 ± 0.005	0.08 ± 0.10	0.13 ± 0.02	0.16 ± 0.15	1.355 0.959 1.914
Thyroid cancer (N, %)	35 (0.18)	0 (0)	336 (0.94)	24 (2.96) ***	3.160 2.074 4.816
Cancer incidence (N, Yes%)	536 (2.68)	1 (1.67)	1461 (4.08)	64 (7.90) ***	1.922 (1.480–2.495)
Asthma (N, Yes%)	279 (1.40)	0(0)	643 (1.80)	20 (2.47)	1.346 (0.857–2.112)
Osteoporosis (N, Yes%)	131 (0.66)	0(0)	2661 (7.43)	92 (11.4) ***	1.381 (1.089–1.752)
Arthritis (N, %)	787 (3.94)	3 (5.00)	3922 (11.0)	109 (13.5) *	1.241 (1.005–1.534)

Values represented adjusted means, standard error-adjusted odds ratios (ORs), and 95% confidence intervals (CI) after adjusting for covariates of age, BMI, residence area, income, education, smoking and drinking status, and physical activity. In analyzing adjusted ORs, each parameter was divided into two groups with the cutoff, which was 25 kg/m^2 1^, 90 cm for men and 85 cm for women ^2^; 100 cm ^3^, 126 mg/dL ^4^, 230 mg/dL ^5^, 160 mg/dL ^6^, 45 for men and 50 mg/dL for women ^8^; 150 mg/dL ^9^, 130 mmHg ^10^, 90 mmHg ^11^, 70 mL/min/1.73m^2 12^, 41 for men and 33 U/L for women ^12^; 40 for men and 32 U/L for women ^13^; and 129 for men and 104 U/L for women ^14^, 4.0 × 10^9^ /L ^15^, 0.5 mg/dL ^16^. ^a, b^ Different superscript letters indicated significantly different at *p* < 0.05. * Significantly different by hyperthyroidism at *p* < 0.05. ** at *p* < 0.01, and *** at *p* < 0.001. ^+^ Significantly different by gender at *p* < 0.05, ^++^ at *p* < 0.01, and ^+++^ at *p* < 0.001. ^#^ Significant interaction between sex and hypothyroidism.

**Table 3:** The characteristics of the ten genetic variants related to an inflammation index used for the generalized multifactor dimensionality reduction analysis.

Chr ^1^	SNP ^2^	Position	Mi ^3^	Ma ^4^	OR ^5^	*p* Value Adjusted ^6^	MAF ^7^	HWE ^8^	Gene	Functional Consequence
1	rs144611984	108270345	A	C	1.91(1.48–2.46)	5.05 × 10^−7^	0.0211	0.4238	*VAV3*	Intron
6	rs7990	32608077	A	C	1.37(1.23–1.54)	6.04 × 10^−8^	0.19	0.0552	*HLA-DQA1*	Missense
6	rs28746784	32635140	T	C	1.48(1.28–1.71)	1.22 × 10^−7^	0.0937	0.6098	*HLA-DQB1*	Nmd transcript
6	rs1800610	31543827	A	G	1.35(1.21–1.52)	2.13 × 10^−7^	0.1921	0.6802	*TNF*	Intron
8	rs11573856	119954995	T	C	0.78(0.68–0.9)	4.74 × 10^−5^	0.1826	0.1248	*TNFRSF11B*	Intron
11	rs11246015	224585	T	C	0.73(0.63–0.85)	5.52 × 10^−6^	0.1482	0.2201	*SIRT3*	intron
12	rs7977554	112882859	A	G	1.55(1.31–1.83)	3.12 × 10^−7^	0.0647	0.0878	*PTPN11*	Nmd transcript
14	rs75664963	81492195	T	A	0.77(0.68–0.86)	7.46 × 10^−6^	0.2704	0.4389	*TSHR*	Intron
15	rs7171366	45386656	G	T	1.45(1.21–1.74)	4.83 × 10^−6^	0.0567	0.1427	*DUOX2*	Intron
15	rs117742123	45429332	T	G	1.49(1.27–1.74)	5.82 × 10^−7^	0.0786	1	*DUOX1*	Nmd transcript

^1^ Chromosome; ^2^ Single nucleotide polymorphism; ^3^ Minor allele; ^4^ Major allele; ^5^ Odds ratio and lower and upper ends of 95% confidence interval; ^6^ *p*-value for OR after adjusting for age, gender, residence area, survey year, body mass index, daily energy intake, education, and income; ^7^ Minor allele frequency; ^8^ *p* value for Hardy–Weinberg equilibrium.

**Table 4:** Adjusted odds ratios for the risk of serum CRP concentrations by the PRS with three SNPs^1^ after covariate adjustments according to age, gender, metabolic syndrome, and nutrient intake.

	Low-PRS (N = 13,856)	Medium-PRS (N = 25,608)	High-PRS (N = 17,200)	Gene–Nutrient Interaction *p* Value
Low WBC ^1^ High WBC	1	2.299 (1.050–5.033) 1.249 (0.942–1.655)	4.887 (2.186–10.93) 1.706 (1.257–2.315)	<0.0001
Low CRP ^2^ High CRP	1 1	1.515 (1.174–1.954) 0.885 (0.306–2.560)	2.221 (1.693–2.915) 0.562 (0.209–1.510)	0.3053
Low EER ^3^ High EER	1 1	1.248 (0.881–1.768) 1.434 (0.945–2.178)	1.893 (1.304–2.748) 2.009 (1.281–3.149)	0.2139
Low CHO ^4^ High CHO	1 1	1.554 (1.088–2.220) 1.238 (0.860–1.782)	2.049 (1.396–3.009) 1.917 (1.297–2.834)	0.0563
Low protein ^5^ High protein	1 1	1.315 (0.963–1.795) 1.449 (0.938–2.237)	1.852 (1.324–2.590) 2.280 (1.438–3.614)	0.2595
Low fat ^6^ High fat 1	1 1	1.285 (0.934–1.770) 1.410 (0.865–2.297)	1.762 (1.246–2.492) 2.367 (1.415–3.958)	0.1768
Low KBD ^7^ High KBD	1 1	1.554 (1.034–2.335) 1.286 (0.912–1.812)	2.654 (1.731–4.067) 1.696 (1.167–2.464)	0.0608
Low PBD ^7^ High PBD	1 1	2.104 (1.184–3.739) 1.161 (0.865–1.558)	2.297 (1.235–4.274) 1.859 (1.357–2.547)	0.0140
Low WSD ^7^ High WSD	1 1	1.168 (0.797–1.712) 1.336 (0.957–1.865)	2.042 (1.360–3.065) 1.797 (1.254–2.577)	0.0295
Low RMD ^7^ High RMD	1 1	1.471 (0.969–2.234) 1.312 (0.944–1.824)	2.271 (1.461–3.531) 1.855 (1.299–2.648)	0.5219
Low PA ^8^ High PA	1 1	1.285 (0.934–1.770) 1.484 (1.020–2.159)	1.762 (1.246–2.492) 2.227 (1.495–3.319)	0.7276
Low coffee ^9^ High coffee	1 1	1.705 (1.088–2.671) 1.104 (0.790–1.541)	2.415 (1.498–3.894) 1.671 (1.165–2.397)	0.1439
Low alcohol ^10^ High alcohol	1 1	1.419 (1.043–1.931) 1.025 (0.597–1.760)	1.962 (1.407–2.737) 1.836 (1.041–3.237)	0.2330
Non-smokers Former and current Smokers	1 1	1.400 (1.089–1.800) 1.687 (0.504–5.645)	2.073 (1.584–2.711) 3.296 (0.952–11.41)	0.0379

Values represent odds ratios and 95% confidence intervals. Gene–gene interaction model with six SNPs included *CRP*_rs386636005, *GUSBP2*_rs1250561232, *OASL*_rs201853167, *APOC1*_rs56131196, *TLDC2*_rs59310406, and *HNF1A*_rs1169286. Low-GRS, medium-GRS, and high-GRS were divided into 0–4, 5–6, and >6 risk alleles for six SNP GMDR models, respectively. The cutoff points of dividing the values of each parameter into two groups were as follows: 4.0 × 10^9^ /L ^1^, 0.5 mg/dL ^2^, estimated energy requirement (EER) ^3^; 70 energy percent (En%) ^4^, 13 En% ^5^, 15 En% ^6^; 66th percentile ^7^, 150 min/week of moderate-intensity physical activity ^8^, 3 cup/week ^9^, and 20 g/day ^10^. Multivariate regression models include the corresponding main effects, interaction terms of gene and main effects (energy and nutrient intake), and potential confounders, such as age, gender, energy intake, residence area, metabolic syndrome, job, education, income, BMI, WBC, smoking, coffee, alcohol, and physical activity. The reference was the Low-PRS. KBD, Korean balanced diet; PBD, Plant-based diet; WSD, Western-style diet; RMD, rice-main diet; PA, physical activity.

## Data Availability

The data were deposited in the Korean biobank (Osong, Republic of Korea).

## Supplementary Materials

The following supporting information can be downloaded at: https://www.mdpi.com/article/10.3390/nu15173850/s1, Figure S1: The distribution of genetic variants related to hypothyroidism; Manhattan plot of genetic variants related to hypothyroidism; Q–Q plot of genetic variants related to hypothyroidism. Table S1: The characteristics of the ten genetic variants of genes in the risk of inflammation used for the generalized multifactor dimensionality. Table S2. Adjusted odds ratios for the risk of hypothyroidism by polygenetic risk scores of the 3 SNPs model (PRS) for gene-gene interaction after covariate adjustments reduction analysis.
