# Supplementary material for: Interactions between Polygenetic Variants and Lifestyle Factors in Hypothyroidism: A Hospital-Based Cohort Study
Source: Nutrients. 2023 Sep 3;15(17):3850. doi: 10.3390/nu15173850 (PMC10490100; doi:10.3390/nu15173850)
Supplement: Supplementary file 1 [file nutrients-15-03850-s001.zip › nutrients-2578774-supplementary.pdf]

Supplementary materials

Figure S1. The distribution of genetic variants related to hypothyroidism

A. Manhattan plot of genetic variants related to hypothyroidism

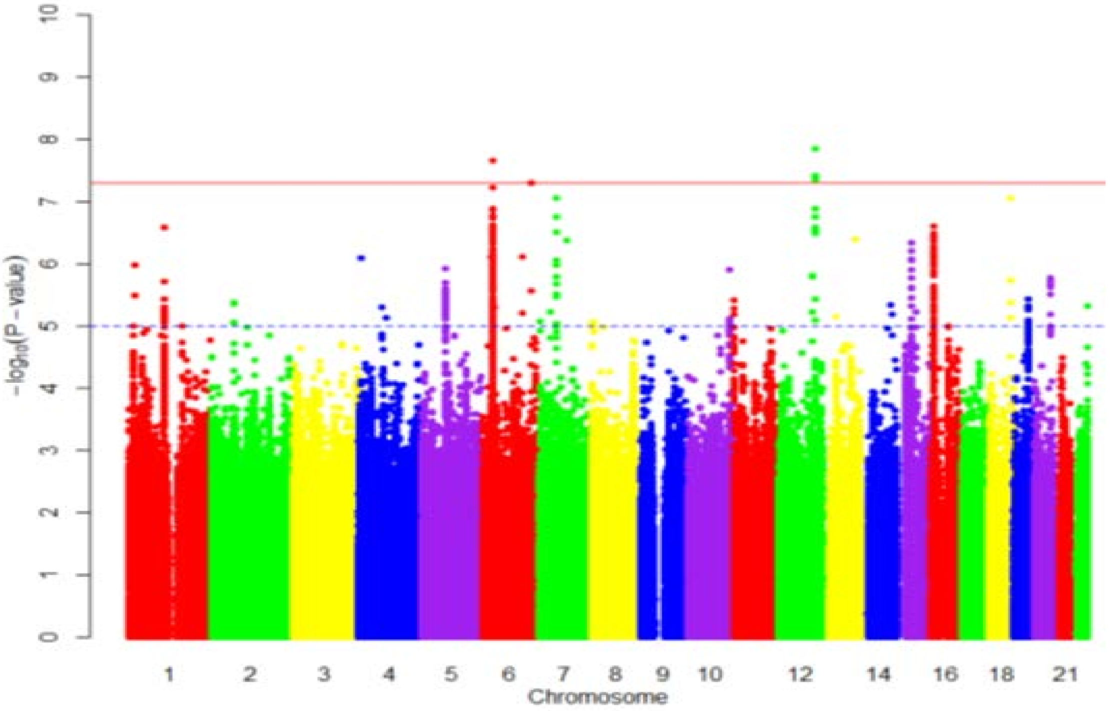

B. Q-Q plot of genetic variants related to hypothyroidism

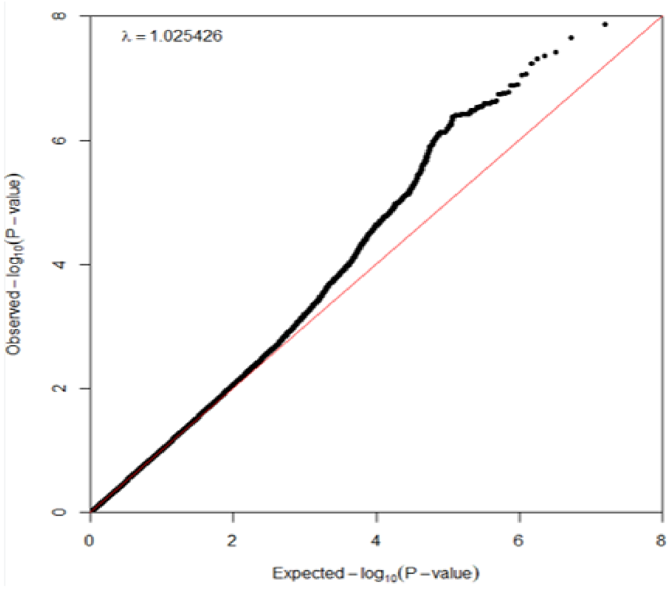

Supplemental Tables

Table S1. The characteristics of the ten genetic variants of genes in the risk of inflammation used for the generalized multifactor dimensionality reduction analysis

Model	Adjusted sex, age, BMI				Adjusted for sex, age, BMI, EER, fat and intake			
	TRBA	TEBA	P value	CVC	TRBA	TEBA	P value	CVC
DUOX1_rs1648314	0.5352	0.5286	8 (0.0547)	9/10	0.5358	0.5295	9 (0.0107)	9/10
TSHR_rs75664963 plus model 1	0.5476	0.5074	6 (0.3770)	3/10	0.5487	0.5119	6 (0.3770)	3/10
**HLA-DQA1_rs17426593 plus model 2**	**0.5668**	**0.5634**	**10 (0.0010)**	**10/10**	**0.5680**	**0.5625**	**10 (0.0010)**	**10/10**
SIRT3_rs11246015 plus model 3	0.5795	0.5391	9 (0.0107)	5/10	0.5806	0.5421	10 (0.0010)	5/10
VAV3_rs4915077 plus model 4	0.6017	0.5566	9 (0.0107)	7/10	0.6027	0.5495	9 (0.0107)	9/10
TNFRSF11B_rs11573856 plus model 5	0.6303	0.5413	10 (0.0010)	5/10	0.6311	0.5341	9 (0.0107)	7/10
**C6orf15_rs2233955 plus model 6**	**0.6699**	**0.5491**	**9 (0.0107)**	**10/10**	**0.6706**	**0.5467**	**9 (0.0107)**	**10/10**
TSHR_rs75664963 plus model 7	0.6971	0.5306	10 (0.0010)	10/10	0.6972	0.5242	9 (0.0107)	10/10
NRG1_rs1487155 plus model 8	0.7190	0.5348	9 (0.0107)	10/10	0.7186	0.5332	9 (0.0107)	10/10
HORMAD2_rs116950391 plus model 9	0.7303	0.5448	9 (0.0107)	10/10	0.7300	0.5410	9 (0.0107)	10/10

CRP, high-sensitive C-reactive protein; *GUSBP2*, glucuronidase, beta pseudogene 2; *HNF-1α,* hepatocyte nuclear factor-1 alpha; *GSTP1*, glutathione S-transferase P-1; *IL1B*, interleukin-1b; NOS2, nitric oxide synthase-2; *C12orf43,* chromosome 12 open reading frame 43; *OASL,* 2'-5'-oligoadenylate synthetase like; *TOMM40*t, ranslocase of outer mitochondrial membrane 40; *APOE*, apolipoprotein E; (rs429358, *APOC1P1*, apolipoprotein C-I pseudogene 1; *TLDC2*, TBC/LysM-associated domain containing 2.

Table S2. Adjusted odds ratios for the risk of hypothyroidism by polygenetic risk scores of the 3 SNPs model (PRS) for gene-gene interaction after covariate adjustments

	Model 1			Model 2	
	Low-PRS (N=13,856)	Medium-PRS (N=25,608)	High-PRS (N=17,200)	Medium-PRS (N=25,608)	High-PRS (N=17,200)
WBC 1	1	1.053 0.955 1.161	1.120 0.998 1.256	1.051 0.953 1.161	1.127 1.004 1.266
CRP 1	1	0.946 0.798 1.121	1.076 0.887 1.305	0.946 0.798 1.122	1.069 0.880 1.299
MetS	1	1.028 0.935 1.130	1.011 0.906 1.128	1.031 0.937 1.134	1.019 0.912 1.138
Osteoporosis	1	0.950 0.828 1.090	0.911 0.775 1.070	0.946 0.824 1.087	0.896 0.761 1.055
Arthritis	1	1.059 0.944 1.188	1.045 0.915 1.194	1.052 0.937 1.181	1.033 0.903 1.181
Asthma	1	1.417 1.067 1.882	1.292 1.001 1.666	1.293 1.001 1.672	1.430 1.075 1.903
Thyroid cancer		0.984 0.712 1.360	1.087 0.751 1.572	0.979 0.708 1.354	1.093 0.755 1.580
Cancer	1	0.868 0.750 1.005	0.937 0.791 1.110	0.869 0.750 1.007	0.938 0.791 1.113
BMI	1	1.060 0.993 1.131	1.008 0.935 1.088	1.059 0.991 1.131	1.008 0.935 1.088
Waist	1	1.017 0.942 1.098	0.953 0.872 1.041	1.009 0.935 1.090	0.948 0.867 1.037
Height		0.969 0.904 1.039	1.019 0.940 1.104	0.968 0.902 1.038	1.014 0.936 1.099
Serum	1	1.008 0.908	1.054 0.935	1.004 0.904	1.057 0.937

glucose		1.118	1.188	1.114	1.192
Serum total cholesterol	1	1.010 0.940 1.086	0.964 0.886 1.048	1.008 0.937 1.084	0.959 0.881 1.043
Serum HDL	1	0.989 0.923 1.060	0.965 0.890 1.046	0.996 0.928 1.068	0.969 0.893 1.051
Serum TG	1	0.970 0.906 1.038	0.943 0.872 1.021	0.972 0.907 1.041	0.944 0.871 1.022
SBP	1	0.989 0.923 1.060	0.965 0.890 1.046	0.935 0.876 1.001	0.932 0.864 1.006
DBP	1	0.960 0.891 1.034	0.946 0.868 1.031	0.960 0.891 1.035	0.949 0.871 1.035

Values represented adjusted odds ratio and 95% confidence intervals.

Gene-gene interaction model with 6 SNPs included *CRP*_rs386636005, *GUSBP2*_rs1250561232, *OASL*_rs201853167, *APOC1*_rs56131196, *TLDC2*_rs59310406, and *HNF1A*_rs1169286. Gene-gene interaction model with 7 SNPs included 6 SNPs in the 6 SNP model plus *C12orf43*_rs2257764.

Low-GRS, medium-GRS and high-GRS were divided into 0-4, 5-6 and >6 risk alleles for 6 SNPs GMDR model, respectively and 0-5, 6-7, >7 risk alleles for 7 SNPs GDMR model, respectively. Number of the participants was for 6 SNP model of serum CRP concentrations.

Covariates of the model 1 was age, genders, BMI, residence areas, education, income, and daily energy intake and those of the model 2 was covariate for model 1 plus smoking and drinking status, exercise, white blood cell counts, seaweed intake, percent fat intake, and percent carbohydrate intake.
